# Identification of potential shared gene signatures between gastric cancer and type 2 diabetes: a data-driven analysis

**DOI:** 10.3389/fmed.2024.1382004

**Published:** 2024-06-06

**Authors:** Bingqing Xia, Ping Zeng, Yuling Xue, Qian Li, Jianhui Xie, Jiamin Xu, Wenzhen Wu, Xiaobo Yang

**Affiliations:** ^1^The Second Clinical College of Guangzhou University of Chinese Medicine, Guangzhou, China; ^2^The Second Affiliated Hospital of Guangzhou University of Chinese Medicine, Guangzhou, China; ^3^State Key Laboratory of Dampness Syndrome of Chinese Medicine, The Second Affiliated Hospital of Guangzhou University of Chinese Medicine, Guangzhou, China; ^4^Guangdong Provincial Key Laboratory of Clinical Research on Traditional Chinese Medicine Syndrome, Guangzhou, China

**Keywords:** bioinformatics, gastric cancer, type 2 diabetes, crosstalk genes, pathways

## Abstract

**Background:**

Gastric cancer (GC) and type 2 diabetes (T2D) contribute to each other, but the interaction mechanisms remain undiscovered. The goal of this research was to explore shared genes as well as crosstalk mechanisms between GC and T2D.

**Methods:**

The Gene Expression Omnibus (GEO) database served as the source of the GC and T2D datasets. The differentially expressed genes (DEGs) and weighted gene co-expression network analysis (WGCNA) were utilized to identify representative genes. In addition, overlapping genes between the representative genes of the two diseases were used for functional enrichment analysis and protein–protein interaction (PPI) network. Next, hub genes were filtered through two machine learning algorithms. Finally, external validation was undertaken with data from the Cancer Genome Atlas (TCGA) database.

**Results:**

A total of 292 and 541 DEGs were obtained from the GC (GSE29272) and T2D (GSE164416) datasets, respectively. In addition, 2,704 and 336 module genes were identified in GC and T2D. Following their intersection, 104 crosstalk genes were identified. Enrichment analysis indicated that “ECM-receptor interaction,” “AGE-RAGE signaling pathway in diabetic complications,” “aging,” and “cellular response to copper ion” were mutual pathways. Through the PPI network, 10 genes were identified as candidate hub genes. Machine learning further selected BGN, VCAN, FN1, FBLN1, COL4A5, COL1A1, and COL6A3 as hub genes.

**Conclusion:**

“ECM-receptor interaction,” “AGE-RAGE signaling pathway in diabetic complications,” “aging,” and “cellular response to copper ion” were revealed as possible crosstalk mechanisms. BGN, VCAN, FN1, FBLN1, COL4A5, COL1A1, and COL6A3 were identified as shared genes and potential therapeutic targets for people suffering from GC and T2D.

## 1 Introduction

As a prevalent tumor worldwide, gastric cancer (GC) has a relatively terrible prognosis. In 2020, estimates from GLOBOCAN revealed that GC ranked fourth for mortality and fifth for morbidity globally (1), which dramatically increased the burden of finance and medical care (2, 3). Type 2 diabetes (T2D) is among the most prevalent endocrine diseases (4). A study representing 215 countries and regions has provided evidence that the global incidence of diabetes will increase, ranging from approximately 10.5% in 2021 to 12.2% in 2045 (5, 6).

T2D is a risk factor for the emergence of certain cancers (7) and is connected to a higher cancer death rate (8, 9). According to cohort research involving over 46,000 patients, diabetes is linked to a 67% increase in GC risk (10). It is also one of the main non-cancer contributors to death in GC (11). Independent of conventional diabetes risk variables, a large-scale cohort investigation has verified that GC is linked to an elevated risk of diabetes (7). As the risk of developing T2D increases by 35% (7) in GC, the mortality of GC survivors also significantly increases (12).

All those empirical observations suggest a strong bidirectional association underlying these two complex diseases. Though the associations between GC and T2D have been widely reported, the underlying genetic processes linking GC and T2D are still unknown. Fortunately, genetic exploration of disease–disease interaction has been possible through advances in sequencing and bioinformatics (4, 13–16). Meanwhile, with the continuous development of machine learning algorithms in medicine, numerous studies have applied them to the screening of feature biomarkers (17–19).

As is well known, exploring shared gene signatures between GC and T2D is of great significance for developing novel therapeutic strategies for joint prediction, prevention, and intervention. Therefore, the goal of our study was to uncover pivotal shared genes and related mechanisms between GC and T2D via bioinformatics and machine learning algorithms.

## 2 Materials and methods

### 2.1 Source of the dataset

“Stomach cancer” and “Type 2 diabetes” were used for digging the Gene Expression Omnibus (GEO) database^1^ for datasets related to either condition. The requirements comprised: (1) *homo sapiens*; (2) each dataset had a sample size of at least 15; (3) the tested tissues were gastric tissue or pancreatic islets; and (4) one disease group and one control group should be included in the dataset.

The annotation soft tables were downloaded from the relevant GPL platform. Probe ID and ensemble ID were converted to official gene symbols through R (v4.2.0) and Perl (v5.30.3), respectively.

### 2.2 Differentially expressed gene screen

GC-related datasets further identified the differentially expressed genes (DEGs) using limma (20) R (version 3.5.1), while T2D-related datasets were analyzed using edge (21) R packages version 3.19. Raw datasets underwent normalization to identify potential mechanisms and relevant biological characteristics associated with pathways of DEGs in GC and T2D. Subsequently, the normalized datasets were transformed using the log2 function. When a replicated gene symbol is mapped to the identical gene, the average expression intensity is taken. DEGs need to meet | logFC| ≥ 1 as well as an adjusted *p*-value of < 0.05. DEGs were shown as heatmaps and volcano plots through ggplot2 (22) and pheatmap (23) packages.

### 2.3 Weighted gene co-expression network analysis

The GC and T2D datasets were subjected to WGCNA through the WGCNA (24) package. First, the goodSamplesGenes function was used to filter qualified samples and genes from the expression matrix to produce a scale-free co-expression. Outliers in the remaining samples were detected using cluster analysis. Next, “pickSoftThreshold” function was utilized to determine the network topology information, and the appropriate β value was selected as the soft threshold to construct the network to make the network meet the scale-free topology characteristics (25). Pearson analysis was used to compute the gene correlations matrix files. Then, the adjacency matrix was constructed by combining the gene correlations matrix and β. In the next step, we first constructed a topological overlap matrix (TOM) by transforming the adjacency matrix, allowing us to assess the gene relationships and dissimilarities within the network. Subsequently, hierarchical clustering was used to group genes based on their similarity in expression profiles, and the dynamic tree-cut function was applied to identify distinct modules within the network. Additionally, we computed module eigengenes (MEs) by evaluating the correlation coefficients between each module and the gene expression patterns. This facilitated the identification of modules strongly associated with specific biological processes (BPs) or conditions within the dataset. Genes exhibiting strong connections within these modules were then selected for further investigation.

### 2.4 Identification of crosstalk genes

DEGs, as well as genes in related ME, were considered representative genes for the disease. Thus, crosstalk genes were defined as genes that overlapped between the GC-related and T2D-related representative genes.

### 2.5 Functional enrichment analysis

The top 10 pathways or BPs were selected based on their significance scores derived from the DAVID website’s^2^ analysis. Specifically, the Gene Ontology (GO) and Kyoto Encyclopedia of Genes and Genomes (KEGG) databases were utilized (26, 27). The enrichment results were then processed using the ggplot2 (22) and stringr (28) packages in the R programming language to generate visualizations. ggplot2 was used for creating graphical representations of the enrichment results, while stringr facilitated data manipulation tasks, such as parsing and formatting pathway names for improved visualization clarity. This approach allowed for the clear visualization of the most relevant pathways and BPs implicated in the analyzed gene expression data.

### 2.6 Protein–protein interaction network

The STRING database^3^ (29) yielded PPI networks for crosstalk genes. The outcomes were then fully visualized using Cytoscape software Version 3.10.2 (30). MCODE with filter criteria including a degree cutoff of 2, a node score cutoff of 0.2, a k-core of 2, and a maximum depth of 100 and cytoHubba (using MCC) plugins were used to screen core gene clusters/hub genes (31–34). Candidate hub genes were defined as the top 10 genes ranked by the cytoHubba plugin and genes in the first cluster chosen by the MCODE plugin.

### 2.7 Machine learning

Support vector machine-recursive feature elimination (SVM-RFE) (35) and least absolute shrinkage and selection operator (LASSO) algorithms were used to select hub genes for GC diagnosis. SVM-RFE minimizes the feature set and identifies predictive features by training a subset of features from various categories using 10-fold cross-validation (36). The SVM-RFE algorithm was implemented for hub gene selection utilizing the kernlab (37, 38), caret (39), and e1071 (40) packages. LASSO regression could increase the predictability and understandability of a statistical model through variable selection (41). LASSO regression was carried out via glmnet (42) package, with a 10-fold cross-verification as the turning/penalty parameter. Hub genes of GC diagnosis were the intersection genes of the two machine learning algorithms.

### 2.8 External validation

To validate hub genes, we obtained clinical and gene data of GC cases from the Cancer Genome Atlas (TCGA) database.^4^ A volcano plot demonstrated differential expression of hub genes, as well as gene expression comparison between cancer groups and control groups, which was further illustrated in a boxplot using reshape2 (43) and ggpubr (44) packages. ROC curves for hub genes were constructed using pROC (45) and ggplot2 (22) packages. Furthermore, clinical data related to GC obtained through the TCGA database were used for survival analysis (46, 47) within the R v4.2.0 environment for survival analysis. Additionally, the survminer package (48) was utilized for visualization purposes. This enabled us to assess the prognostic significance of identified hub genes, in relation to overall survival rates among GC patients.

## 3 Results

### 3.1 Screening of differentially expressed genes

GSE29272 (GPL96 platform, expression profiling by array) and GSE164416 (GPL16791 platform, expression profiling by high-throughput sequencing) met our inclusion criteria. GSE29272 contained 134 GC samples and 134 control samples, while GSE164416 comprised 39 T2D samples and 18 control samples.

**Figure 1:**
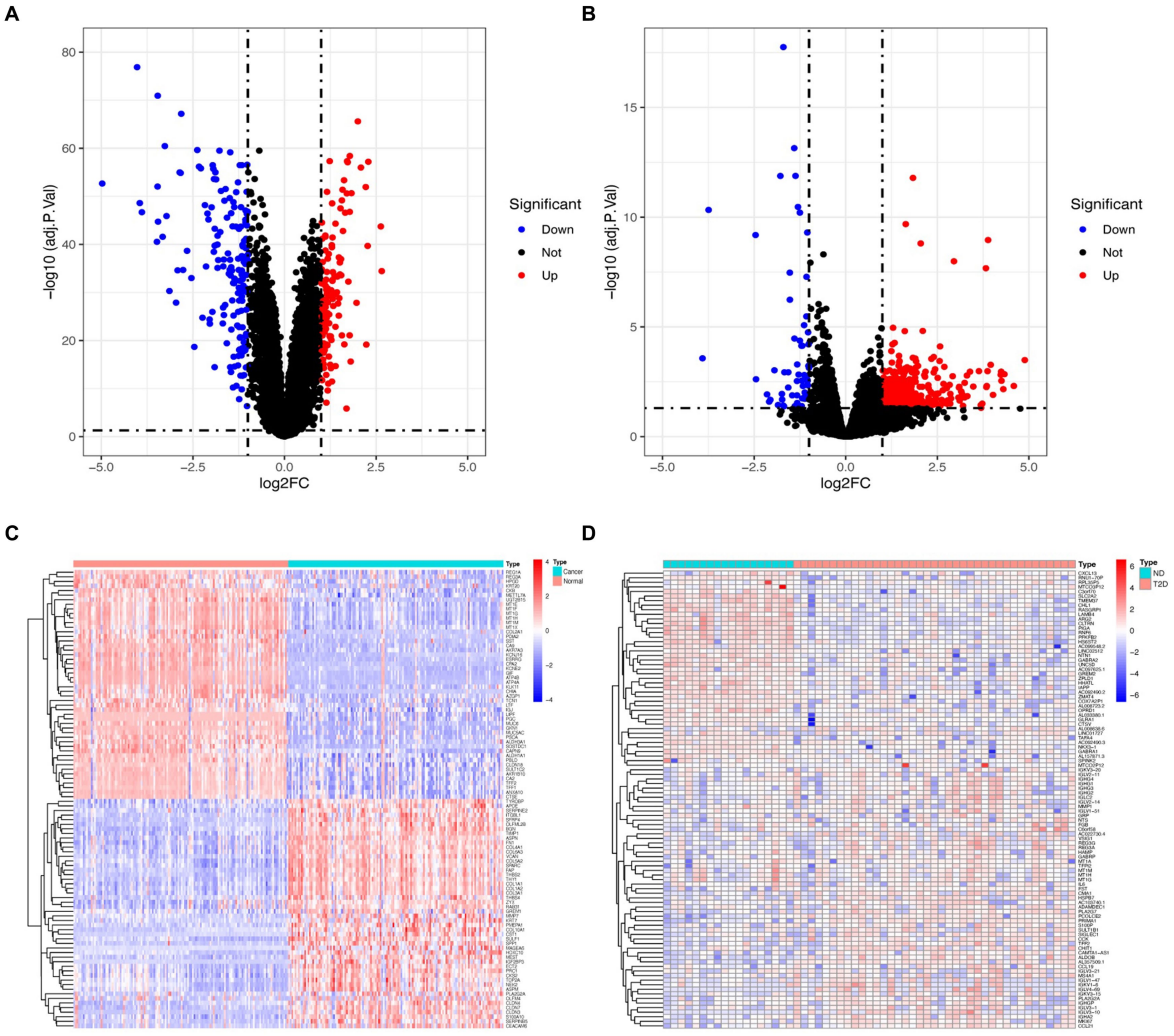
Volcano plot and heatmap for the DEGs identified from GC **(A,C)** and T2D **(B,D)** datasets.

Following the investigation of differential gene expression, 292 DEGs (127 downregulated genes and 165 upregulated genes) were found in the GC dataset, and 541 DEGs (57 downregulated genes and 484 upregulated genes) were discovered in the T2D dataset. Figure 1 displays the volcano plot and heatmaps for DEGs.

### 3.2 Critical module recognition

**Figure 2:**
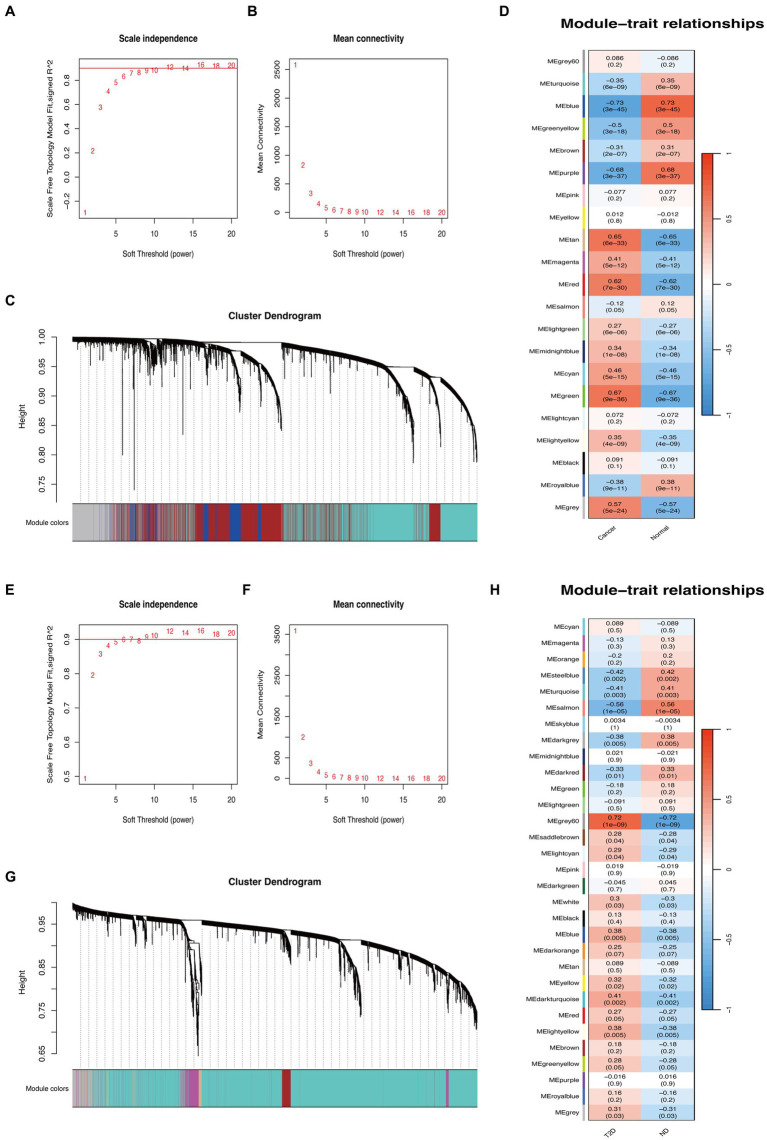
WGCNA of GC and T2D datasets. **(A,B)** β = 8 was chosen as the soft threshold for the GC dataset. **(C)** Cluster dendrogram of co-expressed genes in GC. Under the gene tree, each color represented a module. **(D)** Module–trait relationships in the GC dataset. **(E,F)** β = 6 was chosen as the soft threshold for the T2D dataset. **(G)** Cluster dendrogram of co-expressed genes in T2D. Under the gene tree, each color represented a module. **(H)** Module–trait relationships in the T2D dataset.

Based on WGCNA, 21 modules were discovered in the GSE29272 (β = 8), which were represented in different colors. Through the Spearman correlation coefficient, the “blue” (*r* = −0.73, *p* = 3e-45, 1,863 genes), the “purple” (*r* = −0.68, *p* = 3e-37, 172 genes), and the “green” (*r* = 0.67, *p* = 9e-36, 669 genes) had a high association with GC. Additionally, 31 modules were identified in the GSE164416 (β = 6) with the “grey60” (*r* = 0.72, *p* = 1e-09, 138 genes) and the “salmon” (*r* = −0.56, *p* = 1e-05, 228 genes) having a high association with T2D. The WGCNA results are shown in Figure 2.

### 3.3 Crosstalk genes between GC and T2D

**Figure 3:**
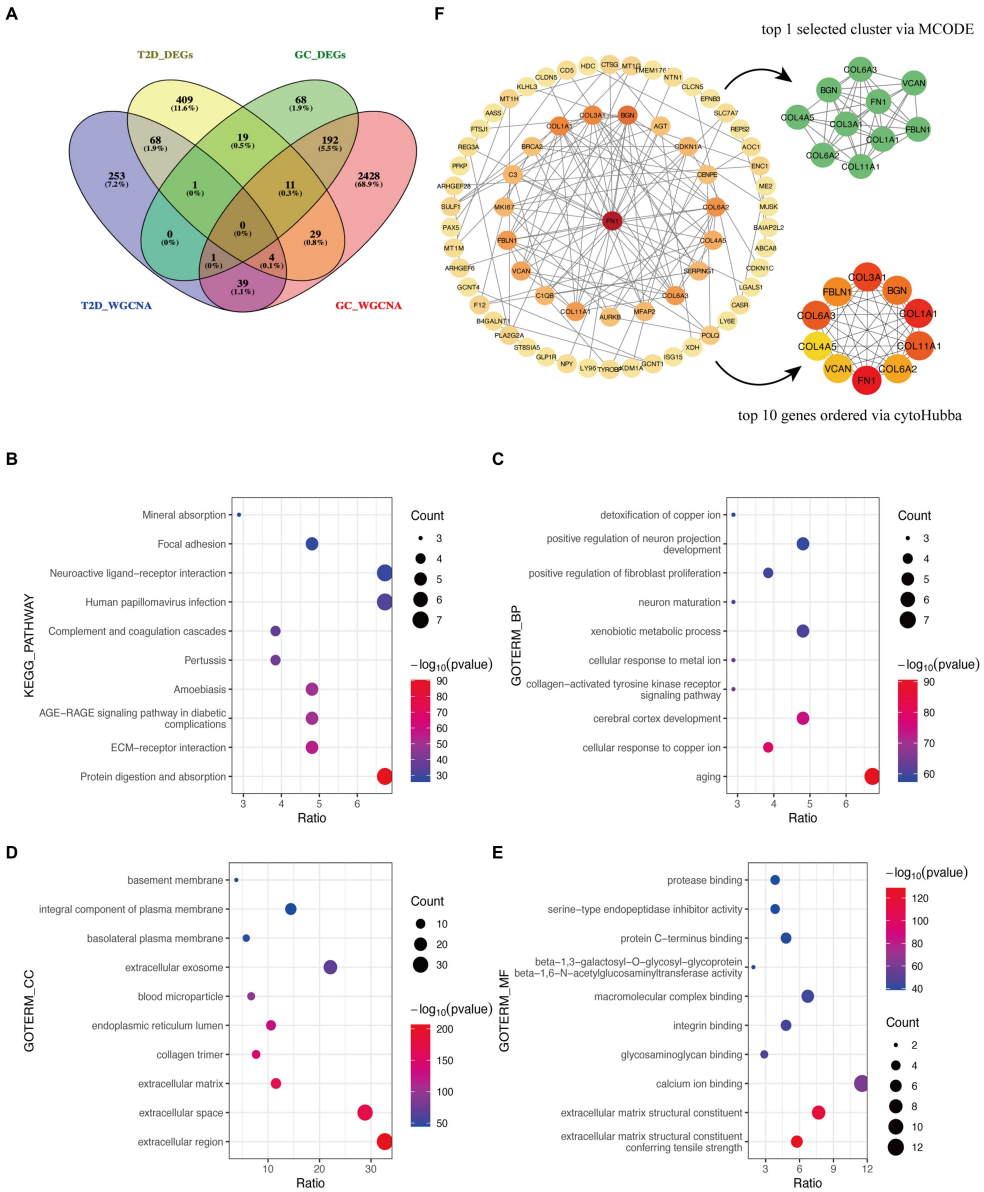
Functional enrichment analysis and PPI network of crosstalk genes. **(A)** The Venn diagram depicts 104 crosstalk genes formed by the intersection of representative genes from the two diseases. **(B)** KEGG analysis. **(C–E)** GO analysis. **(F)** Identification of candidate hub genes.

After the intersection of the representative genes between GC and T2D, 104 genes were identified as the crosstalk genes bridging GC and T2D (Figure 3A; Supplementary Table S1).

### 3.4 Functional enrichment analysis of crosstalk genes

The bulk of crosstalk genes was concentrated in “Protein digestion and absorption,” “ECM-receptor interaction,” and “AGE-RAGE signaling pathway in diabetic complications” through the KEGG analysis (Figure 3B).

In accordance with the GO analysis, the crosstalk genes were primarily enriched in BP categories, including “aging,” “cellular response to copper ion,” and “cerebral cortex development” (Figure 3C). As for cellular component (CC) categories, the crosstalk genes were primarily found in “extracellular region,” “extracellular space,” and “extracellular matrix” (Figure 3D). The three most important molecular functions (MFs) of the crosstalk genes were “extracellular matrix structural constituent conferring tensile strength,” “extracellular matrix structural constituent,” and “calcium ion binding” (Figure 3E). More detailed information is listed in Supplementary Table S2.

### 3.5 Identification of candidate hub genes

**Figure 4:**
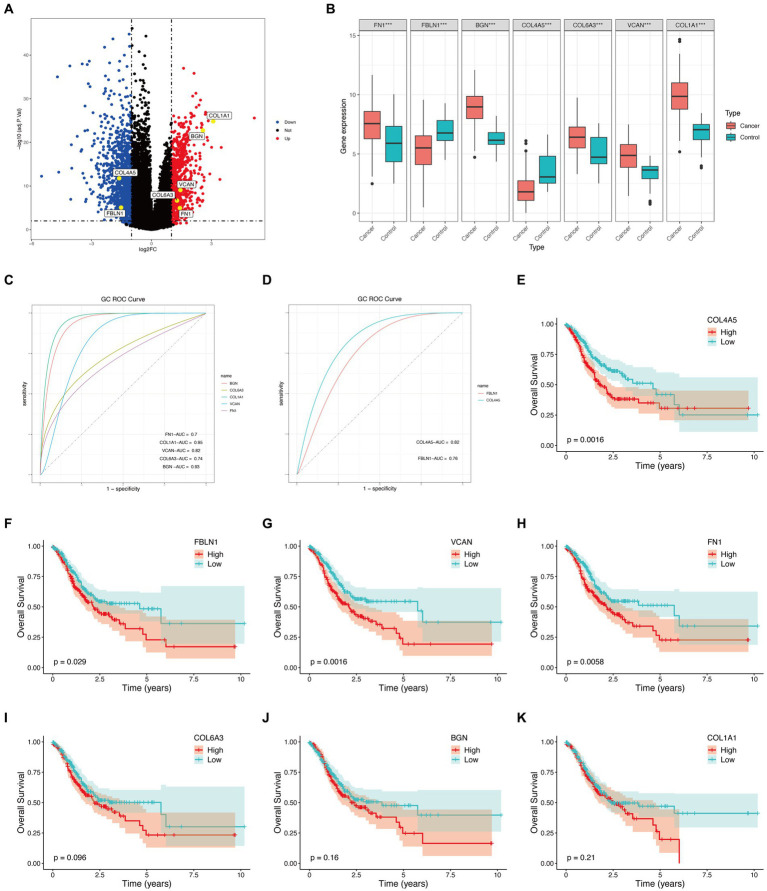
Hub genes external validation and survival analysis in TCGA database. **(A)** Volcano plot of hub genes. **(B)** Boxplot of hub genes (***p < 0.001). **(C)** ROC curves of upregulated genes. **(D)** ROC curves of downregulated genes. **(E–K)** Survival analysis of hub genes.

The PPI network identified 66 interacting node genes and 120 edges in the crosstalk genes for subsequent machine learning filtration (Figure 4F). MCODE and cytoHubba plugins selected 10 genes as closely related genes, respectively. Surprisingly, the 10 genes (FN1, COL1A1, COL3A1, COL6A3, COL11A1, BGN, FBLN1, COL6A2, VCAN, and COL4A5) screened by these two plugins were the same.

### 3.6 Determining hub genes with machine learning

**Figure 5:**
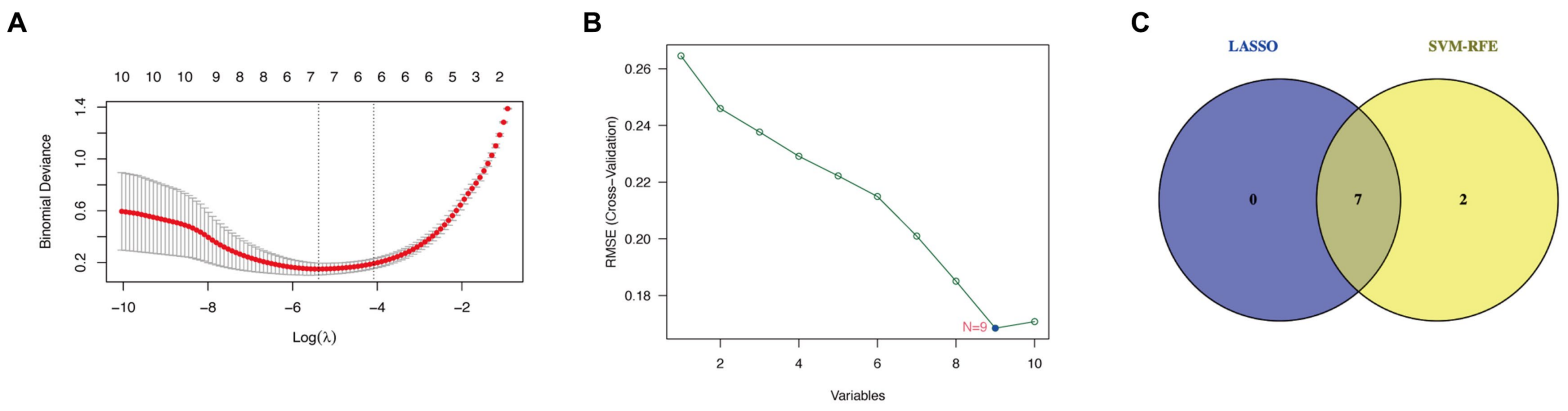
Determining hub genes with machine learning. **(A)** The output of LASSO regression. **(B)** The output of the SVM-RFE algorithm. **(C)** The intersection of the two algorithms is displayed via a Venn diagram.

Seven candidate hub genes were selected through the LASSO regression algorithm (lambda was set to a minimum in order to minimize the regularization effect of the LASSO algorithm) (Figure 5A). In addition, nine candidate hub genes were screened through the SVM-RFE algorithm (Figure 5B). The Venn diagram revealed that the LASSO regression and SVM-RFE algorithms identified seven cross genes (BGN, COL1A1, VCAN, FN1, COL6A3, COL4A5, and FBLN1), which were determined to be hub genes for GC diagnosis in the final validation (Figure 5C).

### 3.7 External validation and survival analysis

The TCGA database consisted of 412 GC samples and 36 control samples. The volcano plot (Figure 4A) and boxplot (Figure 4B) reveal that all seven genes were differently expressed between the two groups. Specifically, COL4A5 and FBLN1 were downregulated genes, while BGN, COL1A1, VCAN, FN1, and COL6A3 were upregulated. Figures 4C,D display the ROC curves corresponding to upregulated and downregulated genes separately. The AUC percentage for BGN, COL1A1, VCAN, FN1, COL6A3, COL4A5, and FBLN1 were 93, 95, 82, 70, 74, 82, and 76%, respectively. The survival analysis of the hub genes detected (COL4A5, FN1, FBLN1, and VCAN) could predict the prognosis of GC (Figures 4E–K).

## 4 Discussion

A meta-analysis revealed that T2D could raise the incidence of GC by 19% (49). In addition, metformin, a common medicine used in the treatment of T2D (50), reduced the risk of GC by 24% (51). Meanwhile, a number of cancer types—particularly those of the pancreas, colon, breast, and stomach—can raise the chance of developing diabetes (7, 52–54). GC and T2D may be co-drivers of each other. The molecular processes driving the complicated interactions between these two diseases remain unexplained. This is the first study investigating the shared genes and common signatures of GC and T2D through bioinformatic analysis and machine learning algorithms in an effort to facilitate early detection, improved treatment, and prompt prevention.

According to the GO analysis, we found the CC and MF of crosstalk genes between GC and T2D were mainly focused on extracellular matrix (ECM), while the KEGG analysis also verified crosstalk genes were enriched in “ECM-receptor interaction.” ECM is a crucial element of the GC microenvironment (55) as well as a major tumorigenesis regulator (56). Excessive deposition of ECM is one of the hallmarks of poor cancer prognosis (57). By stimulating cellular mechanisms associated with cell metabolic control, angiogenesis, and ECM receptors, gastric ECM remodeling promotes the growth of tumors (58, 59). Meanwhile, inappropriate deposition of ECM proteins is associated with multiple complications of diabetes, such as delayed wound healing (60), diabetic retinopathy (61, 62), diabetic foot ulcers (63), and diabetic nephropathy (DN) (64). In addition, hyperglycemia accompanying diabetes can lead to the impaired degradation or synthesis of HSPG in ECM cells, resulting in DN, cardiovascular disease, and retinopathy (65). Therefore, we hypothesized that ECM might be crucial to the comorbidity of T2D and GC.

“Aging” was in first place in the BP of the GO analysis. Cellular senescence is both a cause and a consequence of T2D (66, 67). Numerous senescence-associated secretory phenotypic factors released by senescent cells can mediate the dysfunction of pancreatic β-cell and adipose tissue, as well as insulin resistance, contributing to the pathogenesis of T2D (67, 68). In turn, hyperglycemia and metabolic changes can stimulate senescent cell formation (69), leading to various diabetic complications (70). In addition, senescence contributes to both the prevention and progression of tumors (71). Although senescence has been proven to be a conserved tumor suppressor mechanism (72, 73), the senescence-associated secretory phenotype can also facilitate tumor cell growth (74, 75).

“Cellular response to copper ion” ranked second in BP. Copper ion is associated with the development of T2D and may be a therapeutic target (76). In parallel with this, copper has garnered significant interest in the field of cancer treatment since it may be a limiting element in numerous facets of cancer advancement, such as angiogenesis, proliferation, and metastasis (77, 78). Hence, “cellular response to copper ion” could be the common BP of T2D and GC.

Through KEGG analysis, the “AGE-RAGE signaling pathway in diabetic complications” was considered a bidirectional pathway of T2D and GC. AGEs are synthesized at an accelerated rate during hyperglycemia, and RAGE is an advanced glycation end-product receptor (79). AGEs mainly trigger signaling pathways through RAGE that lead to cellular stress and dysfunction, and harm target organs, resulting in complications (80). Many diabetic complications are associated with AGE-RAGE signaling pathways, such as DN (81), cardiovascular disease (80, 82), and vascular calcification (79). Currently, it has been established that AGE-RAGE signaling contributes to the growth of different cancer (83). AGE-RAGE signaling may facilitate crosstalk between cancer microenvironment components and cells, inducing hypoxia, autophagy, endoplasmic reticulum stress, mitochondrial dysfunction, and epigenetic modification, suggesting that the AGE-RACE signal is an essential driving factor in cancer development (84). According to a study, the AGE-RAGE signaling pathway may generate a positive feedback loop with oxidative stress, increasing the likelihood of cancer in people with diabetes (85).

Through multiple bioinformatics methods, seven hub genes were discovered to be implicated in the co-pathogenesis of GC and T2D. All the hub genes are connected with GC and may be poor prognostic markers in GC and many other cancers (86–92). BGN (93) and FN1 (94) may stimulate GC cell proliferation, invasion, migration, and EMT, which facilitate tumor progression. Aberrant VCAN expression is associated with modifications in ECM homeostasis, cell adhesion, differentiation, and proliferation (95, 96), thereby contributing to the carcinogenic potential of GC (97, 98). FBLN1 is identified as a candidate tumor suppressor gene whose inactivation can contribute to gastric carcinogenesis (89). COL4A5 is an independent prognostic marker for GC, especially in diffuse-type GC (90, 99). COL1A1 was found to be one of the cancer-associated fibroblast (CAF) markers for GC and a poor prognostic signature gene for CAF infiltration (100). COL1A1 is possibly a useful molecular marker and therapeutic target for GC (91). Overexpression of COL6A3 in GC promotes tumor growth and progression (92). It affects the tumor microenvironment, thereby promoting tumor inflammation and angiogenesis (101). As for T2D, FBLN1 is associated with mortality in T2D patients (102). COL1A1 is linked to hypoglycemic activity and is a novel therapeutic target that may be used to treat T2D (103). Although not all hub genes are well understood in T2D, they may be associated with diabetes because they are related to the ECM. VCAN is known to support ECM homeostasis (104). FN1 is an accumulation constituent of the ECM in the case of hyperglycemia (105). FN1 (106), COL1A1 (103) and COL4A5 (107) are significantly correlated with ECM–receptor interaction. COL4A5 (108) and BGN (109) are established to be contributors to the production of excess ECM, regulating ECM deposition. FBLN1 is an ECM protein (110, 111). COL6A3 encodes type VI collagen, which is found in the ECM of practically every tissue (112, 113). Moreover, the hub genes are connected with the development of diabetes complications, such as DN (96, 107, 114, 115), cardiovascular disease (116), and vascular stiffness (117).

In this study, ROC curves suggested that all seven hub genes had a good predictive effect on the occurrence of GC. However, survival analysis revealed that only four genes (VCAN, FN1, FBLN1, and COL4A5) were intimately connected to the GC prognosis. Around the sixth year, there was a reversal in the overall survival rate of the high-risk and low-risk COL4A5 groups. Among them, the overall survival of the high-risk and low-risk groups of COL4A5 was reversed around the sixth year. Nevertheless, GC has a notoriously bad prognosis. According to statistics, less than 20% of patients with advanced cancer infections will survive for 5 years (118). Therefore, we considered that COL4A5 still had good predictive performance. However, further *in vitro* and *in vivo* studies are needed to validate our findings on the functional roles of the identified hub genes in GC. This validation will elucidate molecular mechanisms, validate prognostic significance, and guide the development of diagnostic and therapeutic approaches. Additionally, future research should focus on confirming the prognostic significance of COL4A5 and other genes in independent patient cohorts and exploring their potential as biomarkers for early detection and personalized treatment strategies. These efforts are crucial for translating our findings into clinically relevant applications to improve patient outcomes in GC management.

Despite our speculation on the potential mechanism of the connection between GC and T2D, certain limitations persist. First, only a few corresponding clinical data could be obtained from public databases. In addition, we failed to find patients with both GC and T2D for research. Finally, wet experiments could not be conducted to confirm our results because of the constraints of laboratory conditions.

## 5 Conclusion

This research revealed that “ECM-receptor interaction,” “AGE-RAGE signaling pathway in diabetic complications,” “aging,” and “cellular response to copper ion” are the possible crosstalk mechanisms of GC and T2D. Additionally, it identified seven genes (BGN, VCAN, FN1, FBLN1, COL4A5, COL1A1, and COL6A3) as shared genes and potential targets for treatment in individuals with both GC and T2D. Notably, COL4A5 exhibited a reversal in overall survival rates around the sixth year. However, further investigation is warranted to confirm these conclusions.

## Data availability statement

The original contributions presented in the study are included in the article/Supplementary material, further inquiries can be directed to the corresponding author.

## Author contributions

BX: Conceptualization, Data curation, Funding acquisition, Methodology, Resources, Software, Validation, Visualization, Writing – review & editing. PZ: Conceptualization, Data curation, Methodology, Resources, Software, Supervision, Validation, Visualization, Writing – review & editing. YX: Conceptualization, Data curation, Funding acquisition, Investigation, Resources, Software, Supervision, Validation, Visualization, Writing – review & editing. QL: Data curation, Formal analysis, Investigation, Methodology, Project administration, Writing – review & editing. JXi: Data curation, Formal analysis, Methodology, Project administration, Software, Visualization, Writing – review & editing. JXu: Conceptualization, Formal analysis, Funding acquisition, Methodology, Project administration, Resources, Software, Supervision, Visualization, Writing – review & editing. WW: Conceptualization, Data curation, Formal analysis, Software, Supervision, Validation, Visualization, Writing – review & editing. XY: Conceptualization, Formal analysis, Funding acquisition, Resources, Software, Supervision, Validation, Visualization, Writing – original draft, Writing – review & editing.

## Footnotes

^1^ http://www.ncbi.nlm.nih.gov/geo/

^2^ https://david.ncifcrf.gov/summary.jsp

^3^ https://cn.string-db.org/

^4^ https://portal.gdc.cancer.gov/
